# Predominance of Dystrophinopathy Genotypes in Mexican Male Patients Presenting as Muscular Dystrophy with A Normal Multiplex Polymerase Chain Reaction *DMD* Gene Result: A Study Including Targeted Next-Generation Sequencing

**DOI:** 10.3390/genes10110856

**Published:** 2019-10-29

**Authors:** Miguel Angel Alcántara-Ortigoza, Miriam Erandi Reyna-Fabián, Ariadna González-del Angel, Bernardette Estandia-Ortega, Cesárea Bermúdez-López, Gabriela Marisol Cruz-Miranda, Matilde Ruíz-García

**Affiliations:** 1 Laboratorio de Biología Molecular, Instituto Nacional de Pediatría, Secretaría de Salud, Insurgentes Sur 3700-C, Colonia Insurgentes-Cuicuilco, Alcaldía Coyoacán, 04530 Ciudad de Mexico, Mexico; erandif@yahoo.com (M.E.R.-F.); ariadnagonzalezdelangel@gmail.com (A.G.-d.A.); bernsestandia@yahoo.com.mx (B.E.-O.); cesabelo@hotmail.com (C.B.-L.); 2 Maestría en Ciencias Biológicas, Posgrado en Ciencias Biológicas, Universidad Nacional Autónoma de Mexico, Edificio D, primer piso, Circuito de Posgrados, Ciudad Universitaria, Alcaldía Coyoacán, 04510 Ciudad de Mexico, Mexico; gmcm611@icloud.com; 3 Servicio de Neurología Pediátrica, Dirección Médica, Instituto Nacional de Pediatría, Secretaría de Salud, Insurgentes Sur 3700-C, Colonia Insurgentes-Cuicuilco, Alcaldía Coyoacán, 04530 Ciudad de Mexico, Mexico; matilderuizg@gmail.com

**Keywords:** Duchenne/Becker muscular dystrophies, dilated cardiomyopathy, limb-girdle muscular dystrophies, neuromuscular disorders, Mexican population, next-generation sequencing

## Abstract

The complete mutational spectrum of dystrophinopathies and limb-girdle muscular dystrophy (LGMD) remains unknown in Mexican population. Seventy-two unrelated Mexican male patients (73% of pediatric age) with clinical suspicion of muscular dystrophy and no evidence of *DMD* gene deletion on multiplex polymerase chain reaction (mPCR) analysis were analyzed by multiplex ligation-dependent probe amplification (MLPA). Those with a normal result were subjected to Sanger sequencing or to next-generation sequencing for *DMD* plus 10 selected LGMD-related genes. We achieved a diagnostic genotype in 80.5% (*n* = 58/72) of patients with predominance of dystrophinopathy-linked genotypes (68%, *n* = 49/72), followed by autosomal recessive LGMD-related genotypes (types 2A-R1, 2C-R5, 2E-R4, 2D-R3 and 2I-R9; 12.5%, *n* = 9/72). MLPA showed 4.2% of false-negatives for *DMD* deletions assessed by mPCR. Among the small *DMD* variants, 96.5% (*n* = 28/29) corresponded to null-alleles, most of which (72%) were inherited through a carrier mother. The *FKRP* p.[Leu276Ile]; [Asn463Asp] genotype is reported for the first time in Mexican patients as being associated with dilated cardiomyopathy. Absence of dysferlinopathies could be related to the small sample size and/or the predominantly pediatric age of patients. The employed strategy seems to be an affordable diagnosis approach for Mexican muscular dystrophy male patients and their families.

## 1. Introduction

The X-linked dystrophinopathies [Duchenne (MIM#310200) and Becker (MIM#300376) muscular dystrophies or DMD/BMD] are the most common form of childhood-onset inherited muscular dystrophy. However, there are other muscular dystrophies that are characterized by progressive deterioration of the proximal and/or distal musculature with variable cardiorespiratory compromise and life span; these are generically called “limb-girdle muscular dystrophies” or LGMD. To date, at least 37 LGMD loci have been identified and the disease inheritance patterns classified as autosomal dominant (10 loci) or autosomal recessive (27 loci) [1]. Proper differential diagnosis between the LGMD subtypes and/or between LGMD and dystrophinopathy is essential for accurate medical management, prognosis, genetic counseling and treatment, which can include genotype-based molecular therapies currently under development [2,3,4] or even gene-editing strategies [5]. The advent of next-generation sequencing (NGS) technology has revolutionized the non-invasive and accurate diagnosis of neuromuscular disorders, including muscular dystrophies [1,6].

Large rearrangements (partial intragenic exonic deletions, ~68%; duplications, ~11%) or single nucleotide substitutions/micro*indels* (~20%) in the *DMD* gene (Xp21.2–p21.1, MIM*300377) account for the responsible genotypes of ~99% of DMD/BMD cases [3]. In Mexico, the frequency (52–67.5%) and distribution of partial intragenic deletions at the two major “hot-spots” of the *DMD* gene are well documented [7,8,9]. In one study, partial intragenic duplications accounted for ~10% (*n* = 16/162) of DMD/BMD Mexican patients, 10.5% of cases bore a nonsense single nucleotide variation that could be identified directly by modified multiplex ligation-dependent probe amplification (MLPA), but the responsible genotype was not identified in >30% of patients, as the authors did not sequence the entire coding region or exon-intron borders of the *DMD* gene [9]. Thus, the complete mutational spectrum of DMD/BMD in Mexican patients remains unknown. Moreover, there is limited information in the literature regarding the clinically and genetically heterogeneous group of LGMD patients in the Mexican population [10,11]. An immunodetection study performed on muscle biopsies of 290 patients revealed that, after dystrophinopathies (52.3%), selected LGMD subtypes (i.e., dysferlinopathies, sarcoglycanopathies, calpainopathies and caveolinopathies) accounted for the second largest proportion (33%) of Mexican patients referred with a muscular dystrophy [12]. However, their responsible genotypes were not assessed. Our group recently reported that the *FKRP*-related disorders, which were not evaluated in the study by Gómez-Díaz et al. [12], underlie the genetic etiology of nearly 3% of Mexican patients with a neuromuscular disorder of unknown etiology [13].

Because less than one-third of DMD cases in Mexico are expected to achieve a “definitive” dystrophinopathy diagnosis based on DNA or immunodetection analysis [14], it would be useful to develop an affordable, non-invasive and first-line diagnostic tool that can be used to identify the underlying genetic etiology of clinically suspected muscular dystrophy in Mexican patients without a *DMD* gene deletion identified by the conventional multiplex polymerase chain reaction (mPCR) method. Here, we sequentially applied MLPA followed by Sanger sequencing (SS) of the *DMD* gene, or assessed a targeted NGS gene panel that included the *DMD* gene and 10 selected LGMD-related loci, which yielded a molecular diagnosis in the 80.5% of those cases with suspected muscular dystrophy bearing a previous normal mPCR result.

## 2. Methods

### 2.1. Patients

From our in-house laboratory registry, we selected 72 unrelated male patients (ages available for 58/72; mean age at referral, 11.25 years of age; age range, 2 to 32 years). These individuals were all recruited between 1990 and 2017; they were referred by a pediatric neurologist and/or a clinical geneticist due to a clinical suspicion of dystrophinopathy, but had normal mPCR results for 22 exons representing the two “hot-spots” of the *DMD* gene (Dp427m isoform, NM_004006.2: pm1, 3, 6, 8, 12, 13, 16, 17, 19, 43 to 45, 47 to 55 and 60). Detailed clinical and laboratory data were not available in all cases, but the suspicion of muscular dystrophy was generally based on: (a) proximal weakness (*n* = 35), (b) hyper-creatine-kinase-emia (hyperCKemia, *n* = 44), (c) a myopathic pattern on electromyography (EMG, *n* = 22), (d) dystrophic changes (*n* = 20), and/or (e) altered dystrophin immunoanalysis (*n* = 13) on muscle biopsy. Of the included patients, 41.6% (*n* = 30/72) met three or more of these criteria and at least 33.3% (*n* = 24/72) had a familial history of affected matrilineal male relatives compatible with an X-linked neuromuscular disorder. A single family had genealogy suggestive of an autosomal recessive trait.

In order to perform a genotypic confirmation, assess the pathogenicity criteria of the identified variants and determine the DMD/BMD or LGMD carrier status, we included the mothers of 58 of the 72 patients (genealogies indicated that 24 of them were obligate carriers for an X-linked trait, and one for an autosomal recessive trait), as well as other affected or unaffected relatives. This study was conducted in accordance with the Declaration of Helsinki, and the protocol was approved by the Ethics and Research committees of National Institute of Pediatrics, Mexico (Registry 068/2015).

### 2.2. MLPA Analysis

Uncommon deletions not covered by the mPCR and duplications of 79 exons of the *DMD* gene, along with the alternative promoter/exon 1 of Dp427c isoform, were searched in all 72 patients, using MLPA performed according to the manufacturer’s instructions (SALSA^®^ MLPA^®^ probemix P034-B1 DMD and P035-B1 DMD; MRC-Holland^®^, Amsterdam, The Netherlands). Alterations in the gene dosage of an isolated exon were corroborated by end-point PCR and further SS for single-exon deletions, as well as by real-time PCR (double delta Ct method) [15] or a second independent MLPA assay for single-exon duplications. Identified *DMD* gene deletions or duplications were directly searched by MLPA on female or male relatives for carrier diagnosis and genotype confirmation. Each deletion and duplication was annotated according to Human Genome Variation Society (HGVS) nomenclature and assessed for the resulting reading frame using the *DMD* exonic deletions/duplications reading-frame checker ver. 1.9 at Leiden Muscular Dystrophy pages^©^ [16].

### 2.3. SS of DMD and Targeted NGS Resequencing of DMD and 10 LGMD-Causing Loci

**Figure 1:**
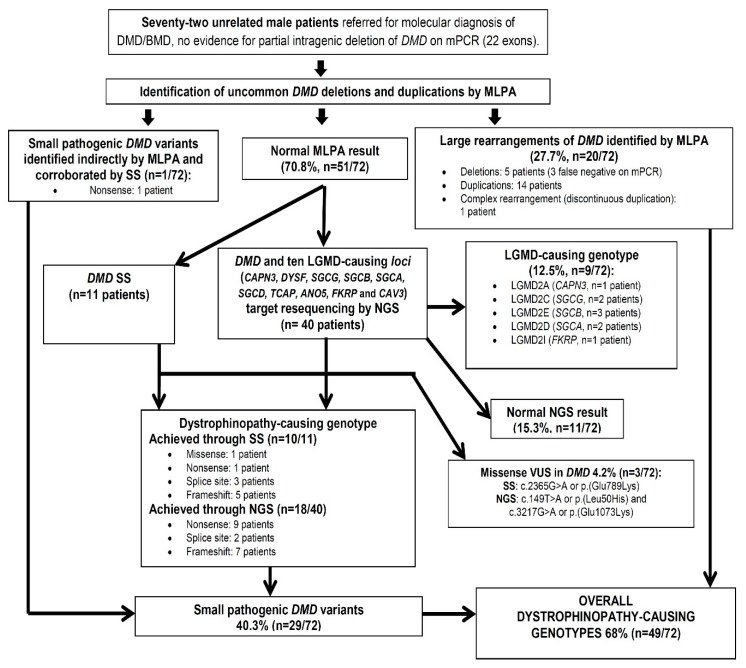
Flow diagram depicting the dystrophinopathy (DMD/BMD) and limb-girdle muscular dystrophy (LGMD) genotypes identified along the different stages of the present study. LGMD2A, 2C, 2E, 2D and 2I, now officially are LGMD R1, R5, R4, R3 and R9 respectively, and according to the LGMD classification proposed by the European Neuromuscular Centre (ENMC) [1]. Abbreviations: MLPA: multiplex ligation-dependent probe amplification; NGS: next-generation sequencing; SS: Sanger sequencing.

Before the availability of the targeted NGS gene panel sequencing, we first selected 11 of 51 patients resulting with a normal MLPA result for SS of *DMD* gene using previously published conditions [17], on basis of an X-linked inheritance (*n* = 3/11), a typical muscle proximal involvement (*n* = 11/11), hiperCKemia (*n* = 10/11), myopathic pattern at EMG (*n* = 3/11), dystrophic changes (*n* = 5/11) and immunohistochemical altered pattern of dystrophin on muscle biopsy (*n* = 6/11). The remaining 40 patients were analyzed using a targeted NGS gene panel containing the following genes: *DMD* (MIM*300377, Xp21.2-p21.1), *CAPN3* (MIM*114240, 15q15.1), *DYSF* (MIM*603009, 2p13.2), *SGCG* (MIM*608896, 13q12.12), *SGCB* (MIM*600900, 4q12), *SGCA* (MIM*600119, 17q21.33), *SGCD* (MIM*601411, 5q33.2-q33.3), *TCAP* (MIM*604488, 17q12.2), *ANO5* (MIM*608662, 11p14.3), *FKRP* (MIM*606596, 19q13.32) and *CAV3* (MIM*601253, 3p25.3) (Figure 1). NGS for *DMD* and the LGMD-related genes was performed by Admera Health (NJ, USA, https://www.admerahealth.com/). The LGMD genes were selected based on their frequencies identified by immunoanalysis of muscle biopsies from Mexican patients with suspected muscular dystrophies (*CAPN3, DYSF, SGCG, SGCB, SGCA, SGCD* and *CAV3*) [12], evidence for a founder effect in our population (*CAPN3* [11] and *FKRP* [13,18,19]), difficulties performing immunological assessment of the encoded protein in muscle biopsies (*ANO5, CAPN3* and *FKRP*) [4,12], inaccuracy in the results of the immunological assessment (*CAPN3*) [20] or evident overlap of clinical manifestations with DMD/BMD, such as the predominance of childhood onset for proximal or lower muscle weakness, calf pseudohypertrophy, hyperCKemia or cardiomyopathy, as in the cases of *TCAP-* or *FKRP*-related disorders and sarcoglycanopathies [1,2,4].

The 11-gene custom NGS panel included all coding exons and intron/exon boundaries. NGS libraries were prepared with KAPA Hyper Prep kit (Kapa Biosystems, Cape Town, South Africa) according to the manufacturer’s protocol. Targets were captured by hybridization with 125-mer probes designed by Twist Bioscience (San Francisco, CA, USA) for 30 nucleotides with 50 × tiling using the hg38 reference genome. Libraries were then sequenced on an Illumina HiSeq2000 2 × 150 platform (San Diego, CA, USA). A posterior in-house bioinformatics pipeline was then applied; it included performing an overall quality evaluation of raw output reads with FastQC v0.11.8 [21], trimming of adapters and filtering of low-quality reads using Trimmomatic v 0.35 [22], alignment of filtered reads against the GRCh38 human reference sequence using Bowtie2 software v2.3.4.1 [23] and calling of single nucleotide variations and detection of small insertion-deletions (*indels*) with the FreeBayes [24] and GATK [25] programs. Variant annotation and filtering (nonsense, frameshift, canonical splice site disruption, start or stop loss, missense and in-frame *indel* changes) was carried out using the Alamut Batch and Alamut Focus software packages (Interactive Biosoftware, Rouen, France), respectively.

The pathogenicity or benignity of the novel missense variants or variants of unknown significance (VUS) was scored according to the standards and guidelines for the interpretation of sequence variants recommended by the American College of Medical Genetics and Genomics and the Association for Molecular Pathology (ACMGG/AMP) [26]. All variants identified by SS or NGS and judged to be clinically relevant to DMD/BMD or LGMD were validated by end-point PCR and SS in the affected cases and their available affected or unaffected relatives. They were then searched in the following: Single Nucleotide Polymorphism Database (dbSNP) [27]; ClinVar [28]; the Human Gene Mutation Database (HGMD) [29]; the DMD Mutations Database, UMD-DMD France [30]; the Genome Aggregation Database (gnomAD) [31]; and the Leiden Open Source Variation Database (LOVD) [32].

## 3. Results and Discussion 

Condensed results are shown in Figure 1, and the genotypic, familial and available phenotypic data of each patient with a confirmed dystrophinopathy or LGMD are summarized in Table 1. The combined MLPA/SS/NGS strategy determined the overall genetic etiology in 80.5% of suspected muscular dystrophy cases bearing a previous normal mPCR result (*n* = 58/72); they included predominantly X-linked dystrophinopathy-related genotypes (68%, *n* = 49/72) followed by LGMD genotypes (12.5%; *n* = 9/72). We also identified 4.1% (*n* = 3/72) of cases with three different missense VUS in the *DMD* gene. We failed to find any pathogenic genotype or VUS in 15.2% (*n* = 11/72) of the analyzed cases (Figure 1).

### 3.1. Identification of DMD Genotypes by MLPA

According to international diagnostic guidelines, MLPA is the first-line study to perform in patients with suspicion of DMD/BMD [3]. The strength of this method was reflected in our study, as it enabled the characterization of an additional 29.2% (*n* = 21/72) of DMD/BMD genotypes in patients with normal mPCR results (Figure 1). Our findings were similar to those described in a sample of European patients with previous normal results on mPCR of 18 exons (32.7%, *n* = 17/52) [33], but higher than those reported in two studies that used the same inclusion criteria with a large group of patients of Hindu descent (11.7%, *n* = 21/180) [34] or in a population of European descent in whom the mPCR assay included 30 exons (15.7%, *n* = 14/89) [35]. 

#### 3.1.1. Partial *DMD* Gene Duplications

We identified apparently contiguous duplications in 19.4% (*n* = 14/72) of cases. This was consistent with previous reports in which large rearrangement in patients with normal mPCR were identified in 17.3% (*n* = 9/52) [33], 11.2% (*n* = 10/89) [35] and 17.7% (*n* = 16/90) [34] of patients from other populations. We also identified a single discontinuous complex rearrangement, for a frequency of 1.4% (*n* = 1/72, patient DMD-1872) in our population. This was consistent with the relevant frequencies reported in European studies, where such rearrangements were found in 1.1% (*n* = 1/89) [35] and 1.9% (*n* = 1/52) [33] of cases.

#### 3.1.2. Partial *DMD* Gene Deletions and mPCR False-Negatives

The proportion of 6.9% deletions herein identified by MLPA (*n* = 5/72) was consistent with this being the second most frequent large rearrangement type identified in DMD/BMD patients with a previous normal mPCR, whose frequencies ranging from 3.3% [34,35] to 13.4% [33]. In the previous studies, infrequent deletions were not initially detected because they involved one or more exons that were not assessed by the employed mPCR assay (mainly located at intermediate region exons 20 to 40 or distal to exon 60). Although this explains the failure of mPCR to detect two of the deletions we identified by MPLA (patients DMD-1302 and -1834), mPCR also failed to detect deletions of exons 45 (DMD-128), 8 (DMD-386, exon deletion 7–9) and 43 (DMD-1355, exon deletion 31–43). This represents a false-negative rate of 4.2% (*n* = 3/72). To the best of our knowledge, this has not previously been reported in the literature, even in a study that involved a higher proportion of infrequent deletions that were not detected by mPCR (13.4%, *n* = 7/52) [33]. Given that the three patients whose deletions were not identified by mPCR were assessed in 1990, 1993 and 2004, we speculate that the false negatives could reflect insufficient standardization of the mPCR technique at the time these studies were conducted and/or subsequent technical improvements in thermal cyclers, the availability of various additives for use in mPCR (i.e., betaine) and/or better performance of recombinant DNA polymerases (i.e., "hot-start" properties). The remaining 69 patients did not show any discrepancy between the mPCR and MLPA results, for a between-method concordance rate of 95.8% (and perfect concordance after 2004). Given that mPCR is faster (yielding results in 4–6 hours), cheaper (it does not require an automated sequencer or the purchase of commercial kits) and technically less demanding, we agree that it could be still considered as an alternative first diagnostic tier for dystrophinopathy (before MLPA or muscular biopsy) in patients of countries with limited resources [3,36,37,38].

#### 3.1.3. Resulting Reading Frame for *DMD* Gene Duplications and Deletions

Only 15% (*n* = 3/20) of the large rearrangements identified by MLPA represented in-frame duplications; the others included out-of-frame duplications (*n* = 11), deletions (*n* = 5) and one complex discontinuous duplication that presented an unpredictable reading frame rule effect, and was identified in a child still ambulant at 12 years of age without corticosteroid therapy (DMD-1872, Table 1). The limited number of patients analyzed precludes us from offering robust conclusions on genotype-phenotype correlations or documenting reading frame rule exceptions, which are expected in 4–9% of dystrophinopathy genotypes [39]. Some of the observed phenotypes appear to be explained (at least in part) by alteration of the dystrophin domain rather than resting solely on a change to the reading frame [3]. In fact, of the three in-frame duplications affecting the critical actin binding domains (encoded by exons 2–10 and 32–45) and the extracellular matrix-interacting domain (encoded by exons 64-70), which are commonly involved in Duchenne phenotypes [3], only one of them was documented in a Becker phenotype (DMD-640). Meanwhile, out-of-frame duplications were identified in patients DMD-1191 (without corticosteroid therapy) and DMD-1751 (deflazacort therapy started at 16 years of age), who were still ambulant at 14 and 17 years of age, respectively.

#### 3.1.4. Indirect MLPA Identification of Small *DMD* Gene Variants

MLPA can indirectly identify point or micro*indel* variants that interfere with the sites of probe hybridization/ligation [35]. We observed this phenomenon only in one hemizygous patient [c.2707G > T or p.(Gly903 *); DMD-1803] for a frequency of 1.4% (*n* = 1/72), which resembled the previously reported figures of 1.1% (*n* = 1/89) [35], 1.9% (*n* = 1/52) [33], and 2.2% (*n* = 2/90) [34]. Unequivocal assignment of the responsible genotype in these cases (including our patient DMD-1803) is of utmost importance for genetic counseling, prenatal diagnosis and the proper selection of whether to offer premature stop codon suppression therapy rather than those based on exon-skipping therapies, which are indicated only for specific *DMD* gene deletions [3].

### 3.2. Small Pathogenic DMD Genotypes Identified through SS and NGS

Easier access to sequencing technology along with growing interest in genotype-based treatments [3] and the development of potential gene-editing therapies [5], has motivated the recent characterization of numerous small pathogenic variants in dystrophinopathy patients bearing a previous normal result on MLPA. Studies have been done in patients of Latin American descent [40,41] and in other countries reporting their first sequencing experiences in the neuromuscular diagnostic setting [38,42]. The diagnosis success rates of these studies have varied widely, from 27% [42] to nearly 100% [40].

Our targeted NGS gene panel generated an output of 26 Mb of read sequences per sample. The coverage statistics showed that the mean sequencing depth in 40 samples was 800X and a 99.99% coverage was achieved for all coding regions and intron/exon boundaries (-20/+20 base pairs) of the 11 studied genes. By combining the SS and NGS data, we identified small *DMD* pathogenic variants in 54.9% (*n* = 28/51) of patients with suspected muscular dystrophy and normal mPCR/MLPA results (Figure 1). This percentage was lower than that obtained through whole-exome sequencing (WES) in the Argentinian setting (84%; *n* = 32/38) [41], where the inclusion criteria were essentially the same as ours, but higher than that achieved in the study reported by Singh B et al. (27.7%, *n* = 5/18) [42], for which limited phenotypic information is available. Other reports with high diagnostic sequencing success for dystrophinopathy in patients with a normal previous MLPA result (nearly 100%, *n* = 104/105) have used more strict inclusion criteria, such as requiring a documented abnormality in the dystrophin immunoanalysis pattern prior to *DMD* gene sequencing [43]. However, the invasiveness of muscle biopsy and secondary abnormalities occasionally seen in the immunohistochemical pattern of dystrophin in patients with *FKRP*-related muscular dystrophies, calpainopathies or sarcoglycanopathies (as noted here in DMD-1421) [4,20,41] may make it difficult to justify performing a muscle biopsy prior to sequencing the *DMD* gene in a given patient. In the present study, 11 of the 13 referred patients with dystrophin abnormalities found on muscle biopsy were found to have a dystrophinopathy genotype, while two had LGMD2D or R3 (DMD-1421) and *DMD* VUS (DMD-1236) genotypes (Table 1).

#### 3.2.1. Small Mutational *DMD* Spectrum 

Of the small *DMD* pathogenic variants herein identified, 96.5% (*n* = 28/29) corresponded to null alleles (frameshift, splice site and nonsense); six of them were previously unreported changes that have been submitted to LOVD. This frequency agrees with those reported in the large TREAT-NMD DMD Global Database (97.9%, *n* = 1415/1445) [39], an Argentinian (100%, *n* = 32/32) [41] and a Spanish populations (93.9%, *n* = 99/106) [43]. Also consistent with the previous studies, we found that single-nucleotide changes represented the predominant small *DMD* mutation type (58.6%, *n* = 17/29 in our study; 71.8–75.23% in the previous reports) [39,41,43]. Despite the small sample analyzed, we also found that one-third of the identified C > T and G > A transitions (*n* = 3/9) were related to CpG dinucleotides [39]. The observed frequency of 37.9% (*n* = 11/29) for premature stop codons resembles that previously reported (~50%) for small pathogenic *DMD* variants [39,43], but it is three-fold higher than the previous data obtained in Mexican patients without exonic deletions/duplications (10.5%, *n* = 6/57) [9], however this difference may reflect the use of a single allele-directed strategy (point mutation-specific MLPA probes) instead of a whole *DMD* gene sequencing. The available clinical information did not suggest any phenotype-genotype discrepancy for null alleles, as none of them were related to Becker phenotypes, and eight such patients (61.5%, *n* = 8/13) were referred with an absence or abnormal pattern of dystrophin immunoanalysis on muscle biopsy (Table 1).

Missense single-nucleotide substitutions are considered to be infrequent dystrophinopathy-causing genotypes that account for less than 2% of these cases [39,40,43]. Most of them abolish the ability of dystrophin to bind to the actin cytoskeleton or to the extracellular matrix through a beta-dystroglycan linkage [3]. The single missense and hypomorphic variant identified in the present study (3.4%, *n* = 1/29) lies inside the N-terminal actin-binding domain (N-ABD) of dystrophin [DMD-1852, c.494A > T or p.(Asp165Val)]. It was previously associated with a Becker phenotype [44], but we cannot establish a definite phenotype correlation for this apparent *de novo* p.(Asp165Val) variant; our 9-year-old patient is still ambulant under deflazacort therapy, has a reported lack of dystrophin on muscle biopsy, but does not have available information regarding which dystrophin epitopes were absent.

#### 3.2.2. Missense VUS in the *DMD* Gene

We found three missense VUS in the *DMD* gene, yielding frequencies of 5.8% (*n* = 3/52) and 4.2% (*n* = 3/72) in the clinically relevant *DMD* genotypes and overall genotypes, respectively. Each VUS was identified in a different family and all three were inherited through a heterozygous mother. However, we lack sufficient clinical data or other available affected or unaffected male relatives to enable definitive pathogenicity/benignity ACMGG/AMP scoring [26]. None VUS were reported in the Spanish setting [43], while this type of allele accounted for 2.6% of patients lacking a clearly pathogenic *DMD* genotype in an Argentinian population examined by WES (*n* = 1/38) [41]. The c.2365G > A or p.(Glu789Lys) [DMD-1313; rs763844939] and c.3217G > A or p.(Glu1073Lys) [DMD-1236, rs398123931] variants are predicted to affect the rod-domain of dystrophin, which is all but devoid of DMD/BMD-causing missense variations [3]. These are extremely low-frequency alleles worldwide according to gnomAD (0.038% and 0.0011%, respectively), although the latter was identified in a patient with a highly suggestive muscular dystrophy phenotype along with “abnormal” dystrophin in muscle biopsy. The p.(Glu1073Lys) has a somewhat higher allelic frequency (0.25%) in Latino populations, where 17 hemizygous individuals are enlisted without any phenotypic information. The third identified VUS, c.149T>A or p.(Leu50His), is absent from the gnomAD database but cataloged as a VUS in ClinVar (RCV000593240.1, RCV000630527.2). The patient harboring this VUS (DMD-1918) met only the PM2, PP3 and BP5 criteria of the pathogenicity/benignity ACMGG/AMP scoring [26], even though the MutationTaster [45], PolyPhen [46], PMut [47], MutPred2 [48], PROVEAN [49] and SIFT [50] programs unanimously predicted this to be a damaging substitution that changes a hydrophobic amino acid (Leu) to a basic residue (His) at a position that shows high phylogenetic conservation (from human to *Drosophila*) and is located inside helix C of the calponin homology type 1 (CH1) domain at N-ABD of dystrophin. Given that nearly 50% of missense dystrophinopathy-causing variants lie in the N-ABD [51], further experimental, phenotypic and segregation evidence are needed to determine whether p.(Leu50His) could exert some deleterious effects on the protein folding, aggregation or actin-binding activity of the mutant dystrophin. Such effects have been documented for the neighboring severe pathogenic variant, p.(Leu54Arg) (rs128626231, RCV000011979.11) [51], which is also located inside helix C of the dystrophin CH1 domain [52].

### 3.3. Mother Carrier Diagnosis for Overall DMD Pathogenic Genotypes

We established the obligate carrier status in 26 (~30% [*n* = 7/26] were initially considered to be isolated cases) of the 44 mothers of molecularly confirmed DMD/BMD patients (Table 1). Heterozygous genotypes in all obligate DMD/BMD carriers were corroborated by MLPA or SS. No genealogic or genotypic data suggesting gonadal mosaicism were noted in any family. The identified deletions and duplications were inherited through a carrier mother in the 50% of cases (*n* = 8/16 available mothers), and we confirmed that in available mothers of affected patients harboring small pathogenic variants of the *DMD* gene had a high risk of being carriers independent of familial history (72% *n* = 18/25). This was consistent with the obligated carrier frequency assumed for small pathogenic *DMD* variants (~85%), wherein is pointed out that such changes tend to arise preferentially during spermatogenesis rather than oogenesis [53].

### 3.4. LGMD Genotypes Identified through NGS 

Five autosomal recessive LGMD subtypes accounted for the genetic etiology of the suspected muscular dystrophy in 12.5% (*n* = 9/72) of all included cases, or 22.5% (*n* = 9/40) of those in which, after a normal mPCR/MLPA result, the genetic etiology of muscular dystrophy was identified by the targeted NGS gene panel. This finding is consistent with the diagnostic rate reported for NGS in Hindu patients presenting as DMD/BMD but with previous normal MLPA results (30%; *n* = 6/18) [42]. Our overall LGMD prevalence is similar to that reported for an Argentinian population analyzed using WES (10.5%, *n* = 4/38), but the latter study unfortunately lacked information regarding zygosity and genotypes for *FKRP*, *SGCG* and *SGCA* [41]. Notably, with the exception of one patient with a *FKRP*-related disorder (DMD-786, presenting as the only example of familial autosomal recessive inheritance), all of the identified LGMD cases had homozygous pathogenic genotypes. These included two previously unreported null genotypes, LGMD2C or R5 (DMD-1762; *SGCG*: c.241_297 + 1169del) and LGMD2D or R3 [DMD-423; *SGCA*: c.696del or p.(Tyr233Thrfs * 15)], which have been submitted to LOVD. The former is a 1226-bp deletion involving portions of exon and intron 3 of the *SGCG* gene; it was identified after a careful re-evaluation of coverage at this locus and thus resembled a female patient in which a homozygous partial exon 6 deletion in the *SGCB* gene was not initially identified by the employed targeted NGS assay (Motorplex) [54]. A later analysis in DMD-1762 by end-point PCR and SS defined the extension and enabled the precise characterization of the breakpoint (NG_008759.1: g.58736_59961del or NM_000231.2(SGCG): c.241_297 + 1169del).

The predominance of homozygous LGMD genotypes suggests that these families could belong to endogamic and/or consanguineous marriages, but this feature only could be confirmed in two of them (the families of DMD-1421 and -1825). We did not identify the founder LGMD2A or R1-causing *CAPN3* pathogenic variant, c.384C > A or p.(Ala116Asp), or the non-founder micro*indel CAPN3* mutation described in patients belonging to an endogamic region of Tlaxcala, Mexico [11]. The previously reported *FKRP* p.[Leu276Ile];[Asn463Asp] heterozygous compound genotype, which to date has been described only in LGMD2I patients of Mexican/Hispanic descent [13,18,19], was herein identified in one non-consanguineous LMGD family (DMD-786), which remarkably was referred with two affected siblings that died during the second and third decade of life; this was attributed to dilated cardiomyopathy, which was corroborated by autopsy in DMD-786. Although cardiomyopathy and other cardiac disturbances are expected in half of LGMD2I or R9 patients [4], dilated cardiomyopathy was not previously described in the five reported Mexican/Hispanic patients bearing the p.[Leu276Ile];[Asn463Asp] *FKRP* genotype [13,19]. However, decreased ejection fractions on echocardiogram were noted for two of these patients (at 21 and 22 years of age) [19], suggesting that the cardiological phenotype for patients with this compound heterozygous *FKRP* genotype has not yet been fully delineated.

#### The Unexpected Absence of Dysferlinopathies

LGMD R2 dysferlin-related (formerly LGMD2B) was the most frequent identified LGMD subtype in a large muscle biopsy immunoanalysis study performed in Mexican patients with suspected muscular dystrophies (18.4%, *n* = 39/212) [12], and is considered the second most common autosomal recessive LMGD form in Brazil [55] as well as certain countries of Asia, Southern and Northern Europe [4,56]. However, we did not identify any patient with dysferlinopathy in the present study. This could be related to our small sample size or the gender bias of our sample (we only included male patients). However, it might also reflect that our patients were mainly characterized by progressive and proximal weakness patterns of childhood onset (mean age at referral: 11.25 years of age; 73% of our patients were < 18 years of age), whereas some allelic forms of LGMD2B or R2 show predominantly early adulthood onset (15–27 years average), subacute polymyositis-like presentation, and distal musculature involvement [56]. This possibility might be supported by the results of Gómez-Díaz et al., as their study population included 36% patients aged ≥ 18 years and 28.9% were affected females, and among the 39 patients identified with dysferlinopathies, the age at diagnosis was 24.29 +/-14.09 years [12]. Although our targeted NGS gene panel assay achieved a deep read of >50X for 99.96% of exonic regions and exon-intron boundaries ( +/-20 bp) of the *DYSF* gene, we cannot discard the possibility of unnoticed heterozygous large gene rearrangements (i.e., exonic deletions) and/or deep-intronic mutations (e.g., NM_003494.3:c.4886 + 1249G > T, RCV000591407.1, rs886042110), which account for to up to 22% of unidentified dysferlinopathy-causing alleles [56].

### 3.5. Comparison of Overall Achieved Muscular Dystrophy Diagnostic Success Rate with other Similar NGS-based Studies

There is currently no clinical consensus regarding the number of loci that should be analyzed by NGS in patients with a neuromuscular disease of unknown etiology. To date, their selection has been rather arbitrary and the diagnostic success has varied widely between studies [6]. For example, by applying two different NGS platforms of 42 and 74 neuromuscular diseases-related genes, Kitamura et al., identified the underlying genetic cause for 60% of 20 patients whose muscular dystrophies had been extensively studied but remained of unknown cause [57]. This is very similar to the results achieved by our application of NGS in muscular dystrophy patients with normal mPCR/MLPA results (67.5%, *n* = 27/40), although our cases had not been so thoroughly evaluated prior to the present study (i.e., no array-CGH, comprehensive/targeted muscle immunoanalysis, mutation searching, or muscle imaging had been performed). In contrast to these frequencies, a NGS study performed in Europe using a commercial targeted gene panel (AmpliSeq Inherited Panel, Life Technologies) covering 325 genes, including LGMD and other muscular dystrophy-related loci, established a genetic diagnosis of LGMD in only 20% of 60 patients clinically cataloged as such [58]. Another study using wide inclusion criteria very similar to ours (hyperCkemia, congenital or early onset of disease, muscle weakness pattern or muscle biopsy results) and NGS evaluation of 65 inherited myopathy-related loci achieved a diagnostic genotype in 41% of 141 patients with muscular dystrophies/myopathies of infantile or juvenile onset [59]. Thus, our study yielded a relatively high diagnostic success rate for NGS (67.5%, *n* = 27/40) compared to the previous reports, even though we analyzed only a small number of muscular dystrophy-related loci (*n* = 11). This may reflect: (a) gender sample bias that favors the identification of dystrophinopathy-related genotypes, especially in male patients at pediatric age (73% of our patients were < 18 years of age); (b) our gene selection criteria, referred to the muscular dystrophy frequencies obtained from muscle immunoanalysis of Mexican patients [dystrophinopathies (52.4%), sarcoglycanopathies (14.1%) and calpaino and caveolinopathies (12.7%)] [12]; and (c) the previous descriptions of LGMD genotypes in Mexican patients [10,11,13,18,19].

Otherwise, we do not reach a molecular diagnosis in 11 patients (Figure 1), mostly of them were at pediatric age (*n* = 7/11) whose myopathy was suspected by proximal weakness (*n* = 4/11), hyperCKemia (*n* = 4/11), myopathic pattern at EMG (*n* = 3/11), dystrophic changes on muscle biopsy (*n* = 2/11) and suggestive family history for an X-linked neuromuscular disease referred in two adult patients; thereby in these last, still remain the possibility (<0.5%) of undetected deep-intronic *DMD* pathogenic changes creating “pseudoexons”, which are only identifiable through dystrophin immunoanalysis and cDNA sequencing at muscle biopsy [3,39,43]. Definite diagnosis in our patients with VUS or normal M-PCR/MLPA and NGS results, could be achieved by a careful clinical re-examination supported by a complete muscle biopsy evaluation or even image studies, in order to corroborate an underlying dystrophic or primary muscle disease, which must be further assessed by a more comprehensive targeted muscle gene panel [6,57,59] or WES [6].

In closing, we suggest that it could be interesting to explore the feasibility of using NGS-based analysis as a first-line diagnostic approach, rather than muscle biopsy [12] or even SS of *DMD* gene, in those Mexican male patients bearing a highly suggestive muscular dystrophy phenotype of early-onset in whom deletions/duplications in the *DMD* gene were previously excluded by MLPA. Our data show that the MLPA/NGS strategy seems to be an affordable diagnostic approach for Mexican muscular dystrophy male patients and their families. Such a strategy has been successfully implemented in other countries (i.e., 76% diagnostic rate for LGMD in Saudi Arabia) [60] that might have limited experience in molecularly diagnosing neuromuscular disease as ours.

## Figures and Tables

**Table 1:** Genotypic and genealogical data from Mexican male patients with a suspicion of muscular dystrophy and normal multiplex PCR results for 22 *DMD* gene exons and further confirmed genetic diagnosis for dystrophinopathy or LGMD.

	Genotype	Patient ID	Available Relevant Clinical Data	NMD Familial History	Relatives’ Genotype Status
	Hemizygous *DMD* gene deletions identified by MLPA ^A^				
1	c.531-?_960 + ?del (DELETION OF EXON 7 TO 9: out-of-frame)	DMD-386	NA. False negative on Multiplex PCR assay (*DMD* exon 8 deletion unnoticed).	ABSENT	Non-carrier mother
2	c.2169-?_2292 + ?del (DELETION OF EXON 18: out-of-frame)	DMD-1834	DMD phenotype (still ambulant at 9 yr), HyperCKemia, MP-EMG.	PRESENT	Carrier mother, two normal homozygous sisters.
3	c.2804-? 4071 + ?del (DELETION OF EXON 22 TO 29: out-of-frame)	DMD-1302	NA	PRESENT	Carrier mother, two healthy hemizygous brothers.
4	c.4234-?_6290 + ?del (DELETION OF EXON 31 TO 43: out-of-frame)	DMD-1355	NA. False negative on Multiplex PCR assay (*DMD* exon 43 deletion unnoticed).	ABSENT	Non-carrier mother
5	c.6439-?_6614 + ?del (DELETION OF EXON 45: out-of-frame)	DMD-128	NA. False negative on Multiplex PCR assay (*DMD* exon 45 deletion unnoticed).	ABSENT	NA
	Hemizygous *DMD* gene duplications identified by MLPA ^A^				
1	c.32-?_93 + ?dup (DUPLICATION OF EXON 2: out-of-frame)	DMD-943	HyperCKemia, dystrophic changes in muscle biopsy.	ABSENT	Non-carrier mother
2	c.32-?_93 + ?dup (DUPLICATION OF EXON 2: out-of-frame)	DMD-1432	NA	ABSENT	Non-carrier mother
3	c.94-?_357 + ?dup (DUPLICATION OF EXON 3 TO 5: in-frame)	DMD-752	BMD phenotype, hyperCKemia.	ABSENT	Non-carrier mother
4	c.94-?_530 + ?dup (DUPLICATION OF EXON 3 TO 6: out-of-frame)	DMD-899	NA	PRESENT	Carrier mother
5	c.94-?_960 + ?dup (DUPLICATION OF EXON 3 TO 9: in-frame)	DMD-640	BMD phenotype, hyperCKemia, MP-EMG, dystrophic changes in muscle biopsy.	ABSENT	NA
6	c.1993-?_2803 + ?dup (DUPLICATION OF EXON 17 TO 21: out-of-frame)	DMD-425	NA	ABSENT	Carrier mother
7	c.4072-?_6290 + ?dup (DUPLICATION OF EXON 30 TO 43: out-of-frame)	DMD-1561	DMD phenotype, hyperCKemia, MP-EMG, dystrophic changes and abnormal immunoanalysis pattern of dystrophin in muscle biopsy.	ABSENT	Non-carrier mother
8	c.4675-?_6290 + ?dup (DUPLICATION OF EXON 34 TO 43: out-of-frame)	DMD-1430	NA	PRESENT	Carrier mother
9	c.6291-?_8217 + ?dup (DUPLICATION OF EXON 44 TO 55: out-of-frame)	DMD-907	NA	PRESENT	Carrier mother and non-carrier sister.
10	c.6615-?_7200 + ?dup (DUPLICATION OF EXON 46 TO 49: out-of-frame)	DMD-1749	DMD phenotype, hyperCKemia, MP-EMG, dystrophic changes in muscle biopsy.	PRESENT	Carrier mother and two carrier sisters.
11	c.7543-?_7660 + ?dup (DUPLICATION OF EXON 52: out-of-frame)	DMD-1191	Still ambulant at 14 yr without corticosteroid therapy, hyperCKemia, MP-EMG, abnormal immunoanalysis pattern of dystrophin in muscle biopsy.	ABSENT	Non-carrier mother
12	c.7543-?_7660 + ?dup (DUPLICATION OF EXON 52: out-of-frame)	DMD-1585	DMD phenotype, hyperCKemia, MP-EMG, dystrophic changes in muscle biopsy.	ABSENT	NA
13	c.7543-?_7660 + ?dup (DUPLICATION OF EXON 52: out-of-frame)	DMD-1751	Still ambulant at 17 yr, hyperCKemia, MP-EMG. Deflazacort therapy initiated at 16 yr.	ABSENT	Non-carrier mother
14	c.8938-?_9807 + ?dup (DUPLICATION OF EXON 60 TO 67: in-frame)	DMD-1460	DMD phenotype	PRESENT	Carrier mother
	Hemizygous *DMD* gene complex rearrangements identified by MLPA^A^				
1	c.[5587-?_7309 + ?dup;9225-?_* 2691 + ?dup] (DISCONTINUOUS DUPLICATION OF EXONS 45 TO 50 AND 63 TO 79: Unpredictable frame rule effect)	DMD-1872	Probable BMD phenotype (still ambulant at 12 yr), hyperCKemia, MP-EMG. No corticosteroid therapy.	ABSENT	NA
	Hemizygous *DMD* genotypes for small variants identified indirectly by MLPA and Sanger sequencing ^B^				
1	c.2707G > T or p.(Gly903 *). LOVD DB-ID: DMD_001252.	DMD-1803	Still ambulant at 8 yr, hyperCKemia. Apparent DMD exon 21 deletion in MLPA assay, which was discarded and correctly annotated by Sanger sequencing.	PRESENT	Carrier mother
	Hemizygous *DMD* genotypes for small variants identified by Sanger sequencing ^B^				
	Missense				
1	c.494A > T or p.(Asp165Val). LOVD DB-ID: DMD_000547	DMD-1852	Still ambulant at 9 yr with deflazacort therapy, hyperCKemia and dystrophin absence by immunoanalysis.	ABSENT	Non-carrier mother
	Non-Sense				
2	c.4758G > A or p.(Trp1586*). ClinVar: RCV000711459.1	DMD-1187	DMD phenotype, hyperCKemia, dystrophic changes in muscle biopsy with dystrophin absence by immunoanalysis.	ABSENT	Carrier mother and normal homozygous sister.
	Splicing				
3	c.2292 + 2T > G. ClinVar: RCV000585714.1	DMD-1372	DMD phenotype, hyperCKemia, MP-EMG.	PRESENT	Carrier mother and affected hemizygous brother; normal homozygous sister.
4	c.2622 + 1G > A. dbSNP: rs398123901	DMD-1890	Still ambulant at 8 yr, hyperCKemia, dystrophic changes in muscle biopsy with dystrophin absence by immunoanalysis.	PRESENT	Carrier mother and affected hemizygous brother.
5	c.7661-1G > A. LOVD DB-ID: DMD_000263	DMD-1793	DMD phenotype, hyperCKemia, dystrophic changes in muscle biopsy with “abnormal” dystrophin pattern by immunoanalysis.	ABSENT	NA
	Frameshift				
6	c.294del or p.(Asp98Glufs * 3) (novel)	DMD-1801	Still ambulant at 9 yr, hyperCKemia, MP-EMG.	ABSENT	NA
7	c.2281_2285del or p.(Glu761Serfs * 10). dbSNP: rs398123881	DMD-1789	DMD phenotype, hyperCKemia, dystrophic changes in muscle biopsy with dystrophin absence by immunoanalysis.	ABSENT	Non-carrier mother
8	c.6128_6131del or p.(Asp2043Valfs * 29). dbSNP: rs863225006	DMD-1837	Still ambulant at 11 yr, hyperCKemia.	PRESENT	Carrier mother
9	c.6446dup or p.(Asp2150Glyfs * 73) (novel)	DMD-1847	Still ambulant at 9 yr, 9 mo; hyperCKemia, dystrophic changes in muscle biopsy with dystrophin absence by immunoanalysis.	ABSENT	Carrier mother
10	c.9204_9207del or p.(Asn3068Lysfs * 20). dbSNP: rs863225015	DMD-1777	DMD phenotype, hyperCKemia, MP-EMG.	ABSENT	Carrier mother
	Variants of unknown significance				
11	c.2365G > A or p.(Glu789Lys). dbSNP: rs763844939	DMD-1313	BMD phenotype (died age 34 yr, unknown cause)	ABSENT	Heterozygous mother. Two normal homozygous sisters.
	Hemizygous *DMD* genotypes for small variants identified by NGS ^B^				
	Non-sense				
1	c.583C > T or p.(Arg195 *). dbSNP: rs398123999	DMD-1395	HyperCKemia.	ABSENT	Non-carrier mother
2	c.2704C > T or p.(Gln902 *). LOVD DB-ID: DMD_003328	DMD-941	NA	ABSENT	Non-carrier mother
3	c.2926G > T or p.(Glu976 *) (novel)	DMD-627	NA	PRESENT	Carrier status confirmed in mother, sister and niece. Hemizygous affected nephew.
4	c.3268C > T or p.(Gln1090 *). ClinVar: RCV000630553.1	DMD-1665	DMD phenotype, hyperCKemia, MP-EMG, dystrophic changes in muscle biopsy with dystrophin absence by immunoanalysis.	ABSENT	Carrier mother
5	c.3274A > T or p.(Arg1092 *). LOVD DB-ID: DMD_000335	DMD-1042	NA	PRESENT	Carrier mother and affected hemizygous brother.
6	c.4757G > A or p.(Trp1586 *). LOVD DB-ID: DMD_000571	DMD-1061	HyperCKemia, dystrophic changes in muscle biopsy.	ABSENT	Non-carrier mother
7	c.5140G > T or p.(Glu1714 *). dbSNP: rs886042747	DMD-1565	HyperCkemia, dystrophic changes in muscle biopsy with dystrophin absence by immunoanalysis.	ABSENT	Carrier mother
8	c.8744G > A or p.(Trp2915 *). HGMD: CM066784	DMD-983	NA	PRESENT	Carrier mother and affected hemizygous brother.
9	c.10171C > T or p.(Arg3391 *). dbSNP: rs398123832	DMD-1884	DMD phenotype.	ABSENT	NA
	Splicing				
10	c.2168 + 1G > T LOVD DB-ID: DMD_046199	DMD-465	HyperCKemia.	ABSENT	Carrier mother
11	c.8937 + 2T > C. LOVD DB-ID: DMD_002149	DMD-757	Died age 33 yr (unknown cause)	PRESENT	Carrier mother and maternal aunt.
	Frameshift				
12	c.1374dup or p.(Glu459Argfs * 4). LOVD DB-ID: DMD_000109	DMD-495	NA	PRESENT	Carrier mother
13	c.2054dup or p.(Thr686Asnfs * 34) (novel)	DMD-1800	Still ambulant at 8 yr, hyperCKemia, MP-EMG.	ABSENT	NA
14	c.2125dup or p.(Gln709Profs * 11) (novel)	DMD-491	NA	ABSENT	Non-carrier mother
15	c.4856_4857del or p.(Lys1619Argfs * 3). HGMD: CD084923	DMD-749	HyperCKemia.	ABSENT	Non-carrier mother
16	c.5864_5886delinsTGAGAGCAAG or p.(Arg1955Leufs * 24) (novel) ^C^	DMD-1579	DMD phenotype, died age 14 yr, hyperCKemia, MP-EMG, dystrophic changes in muscle biopsy with dystrophin absence by immunoanalysis.	PRESENT	Carrier status confirmed in mother and half-sister. Normal homozygous maternal aunt.
17	c.8374_8375del or p.(Lys2792Valfs * 5). dbSNP: rs398124070	DMD-1531	HyperCKemia.	PRESENT	Carrier mother
18	c.10453del or p.(Leu3485 *). dbSNP: rs886043375	DMD-1451	DMD phenotype, hyperCKemia.	PRESENT	Carrier status confirmed in mother and two sisters.
	Variants of unknown significance				
19	c.149T > A o p.(Leu50His). ClinVar: RCV000630527.2	DMD-1918	Proximal muscle weakness, still ambulant at 3 yr, hyperCKemia, MP-EMG.	ABSENT	Heterozygous mother
20	c.3217G > A or p.(Glu1073Lys). dbSNP: rs398123931	DMD-1236	DMD phenotype, hyperCKemia, MP-EMG, dystrophic changes in muscle biopsy with “abnormal” dystrophin pattern by immunoanalysis.	ABSENT	Heterozygous mother and sister.
	LGMD (type and phenotype MIM number) genotypes identified by NGS^B^				
1	*CAPN3* (LGMD2A or R1, MIM#253600): Homozygous NM_000070.2:c.2290del or p.(Asp764Thrfs * 12). dbSNP: rs886044527	DMD-945	“BMD phenotype”.	ABSENT	Carrier mother and sister. Father NA.
2	*FKRP* (LGMD2I or R9, MIM#607155): Compound Heterozygous NM_001039885.2:c.[826C > A];[1387A > G] or p.[Leu276Ile];[Asn463Asp]. dbSNP: rs28937900 and rs121908110, respectively.	DMD-786	“BMD phenotype”, hyperCKemia, MP-EMG and died at age 15 yr due to dilated cardiomyopathy confirmed by *post-mortem* study.	PRESENT (suggestive for autosomal recessive muscular dystrophy trait)	Carrier mother p.[Asn463Asp]; [=]; father NA. One compound heterozygous sister also deceased at 30 yr due to dilated cardiomyopathy.
3	*SGCA* (LGMD2D or R3, MIM#608099): Homozygous NM_000023.2: c.229C >T or p.(Arg77Cys). dbSNP: rs28933693	DMD-1421	“BMD phenotype”, hyperCKemia, MP-EMG, dystrophic changes in muscle biopsy with “abnormal” dystrophin pattern by immunoanalysis.	ABSENT	Obligate carrier status confirmed in both parents (first-grade cousins) and one sister. Two normal homozygous siblings.
4	*SGCA* (LGMD2D or R3, MIM#608099): Homozygous NM_000023.2: c.696del or p.(Tyr233Thrfs * 15) (novel)	DMD-423	NA	ABSENT	NA
5	*SGCB* (LGMD2E or R4, MIM#604286): Homozygous NM_000232.4:c.323T > G or p.(Leu108Arg). dbSNP: rs104893870	DMD-1825	Still ambulant at 11 yr, hyperCKemia, dystrophic changes in muscle biopsy.	PRESENT	Carrier mother and one affected homozygous brother. Non-consanguineous parents, but they came from an inbreeding community (~700 inhabitants, Ejutla, Oaxaca, Mexico).
6	*SGCB* (LGMD2E or R4, MIM#604286): Homozygous NM_000232.4: c.499G > A or p.(Gly167Ser). dbSNP: rs779516489	DMD-621	HyperCKemia.	PRESENT	Carrier mother; father NA. Homozygous affected brother.
7	*SGCB* (LGMD2E or R4, MIM#604286): Homozygous NG_008891.1(NM_000232.4): c.622-2A > G. dbSNP: rs780596734	DMD-954	HyperCKemia, dystrophic changes in muscle biopsy.	ABSENT	Carrier mother; father NA.
8	*SGCG* (LGMD2C or R5, MIM#253700): Homozygous NG_008759.1(NM_000231.2): c.241_297 + 1169del (partial deletion of exon and intron 3, novel)^D^	DMD-1762	“DMD phenotype”, hyperCKemia, MP-EMG. Bilateral retinoblastoma.	ABSENT	NA. Family history of hereditary retinoblastoma.
9	*SGCG* (LGMD2C or R5, MIM#253700): Homozygous NM_000231.2: c.752del or p.(Thr251Serfs * 29). dbSNP: rs886042749	DMD-820	HyperCKemia.	PRESENT	Carrier mother; father NA.

**^A^** Human Genome Variation Society (HGVS) nomenclature and predicted frame rule effect for *DMD* gene deletions and duplications according to LRG_199t1 and NM_004006.2 reference sequences. **^B^** If available, dbSNP, ClinVar, HGMD, or LOVD entries for each *DMD* or LGMD-causing variant are displayed. Novel or previously non-reported variants in the main genotypic databases (dbSNP, ClinVar, HGMD, LOVD, *The DMD mutations database UMD-DMD France*, and gnomAD) are indicated. **^C^** Pathogenic *DMD* gene micro*indel* initially described by NGS as NM_004006.2: c.5864_5876del or p. (Arg1955Leufs * 24), but further correctly annotated by Sanger sequencing. **^D^** Homozygous partial deletion initially detected by NGS due to a very low-depth read at exon-intron 3 of the *SGCG* gene, but further confirmed with the breakpoint delineation by end-point single PCR and Sanger sequencing. **Abbreviations:** BMD: Becker muscular dystrophy; DMD: Duchenne muscular dystrophy; LGMD: limb-girdle muscular dystrophy (legacy and proposed ENMC names are provided) [1]; MIM: Online Mendelian Inheritance in Man; MLPA: multiplex ligation-dependent probe amplification; mo: months; MP-EMG: myopathic pattern in electromyography; NGS: next-generation sequencing; NA: not available; NMD: neuromuscular disorders; PCR: polymerase chain reaction; yr: years.
